# Experimental mouse model of optic neuritis with inflammatory demyelination produced by passive transfer of neuromyelitis optica-immunoglobulin G

**DOI:** 10.1186/1742-2094-11-16

**Published:** 2014-01-27

**Authors:** Nithi Asavapanumas, Julien Ratelade, Marios C Papadopoulos, Jeffrey L Bennett, Marc H Levin, Alan S Verkman

**Affiliations:** 1 Department of Medicine and Physiology, University of California, 1246 Health Sciences East Tower, San Francisco, CA 94143-0521, USA; 2 Academic Neurosurgery Unit, St. George’s, University of London, London SW17 0RE, UK; 3 Departments of Neurology and Ophthalmology, University of Colorado Denver, Aurora, CO 80045, USA; 4 Department of Ophthalmology, University of California, San Francisco, CA 94143, USA

**Keywords:** NMO, Neuroinflammation, Mouse models, Aquaporin, Astrocyte

## Abstract

**Background:**

Although optic neuritis (ON) is a defining feature of neuromyelitis optica (NMO), appropriate animal models of NMO ON are lacking. Most NMO patients are seropositive for immunoglobulin G autoantibodies (NMO-IgG) against the astrocyte water channel aquaporin-4 (AQP4).

**Methods:**

Several approaches were tested to develop a robust, passive-transfer mouse model of NMO ON, including NMO-IgG and complement delivery by: (i) retrobulbar infusion; (ii) intravitreal injection; (iii) a single intracranial injection near the optic chiasm; and (iv) 3-days continuous intracranial infusion near the optic chiasm.

**Results:**

Little ON or retinal pathology was seen using approaches (i) to (iii)*.* Using approach (iv), however, optic nerves showed characteristic NMO pathology, with loss of AQP4 and glial fibrillary acidic protein immunoreactivity, granulocyte and macrophage infiltration, deposition of activated complement, demyelination and axonal injury. Even more extensive pathology was created in mice lacking complement inhibitor protein CD59, or using a genetically modified NMO-IgG with enhanced complement effector function, including significant loss of retinal ganglion cells. In control studies, optic nerve pathology was absent in treated AQP4-deficient mice, or in wild-type mice receiving control (non-NMO) IgG and complement.

**Conclusion:**

Passive transfer of NMO-IgG and complement by continuous infusion near the optic chiasm in mice is sufficient to produce ON with characteristic NMO pathology. The mouse model of NMO ON should be useful in further studies of NMO pathogenesis mechanisms and therapeutics.

## Background

Neuromyelitis optica (NMO) is an autoimmune inflammatory disease of the central nervous system that causes demyelinating lesions in optic nerve and spinal cord, leading to loss of visual and motor function [1-3]. A specific feature of NMO is the presence of serum immunoglobulin G (IgG) autoantibodies (NMO-IgG) against astrocyte water channel aquaporin-4 (AQP4) [4,5]. NMO pathogenesis is thought to involve NMO-IgG binding to AQP4 on astrocytes, which causes complement- and cell-mediated astrocyte cytotoxicity, inflammation, and blood–brain barrier (BBB) disruption, with secondary oligodendrocyte and neuron damage [6-8]. Current therapies of NMO include general immunosuppression, B-cell depletion and plasma exchange [9,10].

Although optic neuritis (ON) with permanent loss of vision is a major clinical feature of NMO [11-13], adequate models of NMO ON are lacking. The particular sensitivity of the optic nerve in NMO suggests the need to study disease mechanisms and treatment responses in optic nerve-specific NMO models. Disease-relevant animal models of NMO are important for investigating pathogenesis mechanisms, such as the role of inflammatory effector cells [14-16] and for testing of potential therapeutics such as antibodies targeting AQP4 [17] or complement [18,19]. The original models of NMO involved administration of NMO-IgG to rats with pre-existing neuroinflammation produced by experimental autoimmune encephalomyelitis, in which immunization with a myelin oligopeptide produces an anti-myelin T-cell response [20-22]. Subsequently, a passive-transfer mouse model of NMO involving intracranial injection of NMO-IgG and human complement recapitulated key pathological findings in NMO, including loss of AQP4 and glial fibrillary acidic protein (GFAP) immunoreactivity, granulocyte and macrophage infiltration, vasculocentric deposition of activated complement, and demyelination [23]. However, due in part to the limited diffusion of AQP4-IgG and complement from the injection site, pathology in this model was confined to a small region around the injection site, sparing the optic nerves.

The purpose of this study was to establish an animal model of NMO ON involving passive transfer of NMO-IgG with targeted delivery to the optic nerves. Mice were chosen for these studies because of the availability of relevant knockout strains (AQP4 and CD59). After testing various approaches we established the conditions in which delivery of NMO-IgG to optic nerves produced ON with characteristic NMO pathology.

## Methods

### Mice

*In vivo* studies were performed on 8- to 10-week-old, weight-matched AQP4^+/+^ and AQP4^-/-^ mice in CD1 genetic background, which were generated as described previously [24]. Some experiments were done on CD59^+/+^ and CD59^-/-^ mice on a C57bl/6 background (provided by Dr Xuebin Qin, Harvard University, USA). Littermates were used as wild-type controls for the AQP4 and CD59 knockout mice. Mice were maintained in air-filtered cages and fed normal mouse chow in the University of California, San Francisco (UCSF) Animal Care facility. All procedures were approved by the UCSF Committee on Animal Research.

### Neuromyelitis optica (anti-aquaporin-4) antibodies

Recombinant monoclonal NMO antibody rAb-53 (referred to as NMO-IgG) was generated from a clonally expanded plasma blast population from cerebrospinal fluid of an NMO patient, as described and characterized previously [22,25]. Purified rAb-53 was used for studies here because of its high affinity for AQP4, and to eliminate the potential variability introduced by using NMO patient serum, which is polyclonal and may contain other antibodies or soluble factors that influence NMO pathogenesis. A NMO ‘superantibody’ with enhanced complement-dependent cytotoxicity (referred as NMO-IgG^CDC+^) was generated as described previously [26] by introducing mutations (G236A/S267E/H268F/S324T/I332E) in the Fc portion of rAb-53 [27].

### Neuromyelitis optica immunoglobulin G antibody delivery to anterior optic nerve and retina

Adult mice were anesthetized with intraperitoneal tribromoethanol (avertin, 250 to 500 mg/kg). Lateral canthotomy was done under a dissecting microscope. Ocular muscles were retracted and anterior optic nerve was exposed to infuse locally 1 μg NMO-IgG and 0.5 μL human complement (Complement Technology, Tyler, TX, USA) in a total volume of 1.5 μL. For intravitreal injection, a 32-gauge needle attached to a 10-μL gas-tight Hamilton syringe was passed through the sclera, next to the limbus, into the vitreous cavity. NMO-IgG (1 or 3 μg) and 0.5 μL human complement in a total volume of 2 μL was injected (0.5 μL per minute) above the optic nerve head.

### Neuromyelitis optica immunoglobulin G antibody delivery to posterior optic nerve

Adult mice were anesthetized and mounted on a stereotaxic frame. A midline scalp incision was made and a burr hole of diameter 1 mm was drilled in the skull 1-mm right and 1-mm anterior to bregma. For single administration of NMO-IgG, a 30-gauge needle attached to a 50-μL gas-tight syringe was inserted through the brain (6 mm below the dura down to base of the skull) near the optic chiasm to deliver 5 μg NMO-IgG and 5 μL human complement in a total volume of 10 μl. For continuous administration of NMO-IgG, an osmotic minipump (Alzet 1003D, Cupertino, Ca, USA) delivered 3.3 μg NMO-IgG and 16.7 μL human complement per day for 3 days.

### Immunofluorescence

Optic nerves were post-fixed for 2 hours in 4% paraformaldehyde. Ten micrometer-thick frozen sections were immunostained at room temperature for 1 hour with antibodies against AQP4 (1:200, Santa Cruz Biotechnology, Santa Cruz, CA, USA), GFAP (1:100, Millipore, Temecula, CA, USA), myelin basic protein (MBP; 1:200, Santa Cruz Biotechnology), ionized calcium-binding adaptor molecule-1 (Iba1; 1:1,000; Wako, Richmond, VA, USA), albumin (1:200, Santa Cruz Biotechnology), C5b-9 (1:100, Santa Cruz Biotechnology), neutrophil (Ly-6G, 1:100, Santa Cruz Biotechnology), eosinophil (siglec-F, 1:50, BD Biosciences, Oxford, UK), macrophage (F4/80, 1:100, Santa Cruz Biotechnology) or CD45 (1:10, BD Biosciences) followed by the appropriate fluorescent secondary antibody (1:200, Invitrogen, Grand Island, NY, USA). Immunofluorescence was examined with a Leica (Wetzlar, Germany) DM 4000 B microscope or Nikon (Melville, NY, USA) laser-scanning confocal microscope. Areas were defined by hand and quantified using ImageJ software (National Institutes of Health).

### Retinal ganglion cell labeling

Retinal ganglion cells (RGCs) were labeled as described previously [28-30]. Briefly, mice were injected with 1 μL neurotracer dye FluoroGold (4% solution in saline; Fluorochrome, Denver, CO, USA) in the superior colliculus (from the bregma, anterior-posterior, -3 mm; medial-lateral, +0.5 mm; 2 mm below the dura) 7 days before NMO-IgG and human complement delivery to posterior optic nerve. Retinas were flattened 14 days after the FluoroGold injection and whole-mounts were fixed in 4% paraformaldehyde. RGCs were counted manually under a fluorescence microscope with a 40× objective. A total of 16 to 20 images per retina were used for cell counting. As a positive control for RGC loss, optic nerve crush injury was produced by 30-second compression of the optic nerve 2 mm posterior to the globe insertion using cross-action forceps. The optic nerve was accessed by lateral canthotomy and dissection beneath the lateral rectus. RGCs were quantified 7 days after crush.

### Statistical analysis

Values are presented as mean ± SEM. Comparisons between two groups were performed using the unpaired Student's *t*-test. *P* < 0.05 was considered statistically significant.

## Results

### Passive transfer of neuromyelitis optica immunoglobulin G antibody and complement via retrobulbar infusion, intravitreal injection or single perichiasmal injection does not produce optic neuritis

**Figure 1:**
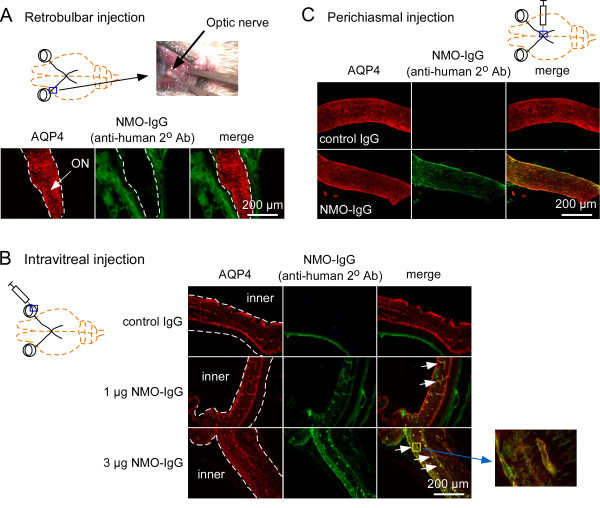
**Passive transfer of neuromyelitis optica immunoglobulin G antibody and complement via retrobulbar infusion, intravitreal injection or single perichiasmal injection does not produce optic neuritis. (A)** (Top) Lateral canthotomy was done in mice to expose the optic nerve (arrow). (Bottom) Absence of binding of neuromyelitis optica immunoglobulin G antibody (NMO-IgG) (as seen using an anti-human secondary antibody) to aquaporin-4 (AQP4) in the optic nerve (dashed line) 3 days after retrobulbar infusion of NMO-IgG and human complement (representative of three eyes from separate mice). **(B)** Binding of NMO-IgG to AQP4 in retina (dashed line) after intravitreal injection. No binding was seen with a control (non-NMO) IgG (n = 5 eyes). (Insert) Magnified view of NMO-IgG binding to AQP4 at perivascular end-feet of inner retinal Müller cells. **(C)** Binding of NMO-IgG (and not of control IgG) to AQP4 after single perichiasmal injection. Despite efficient binding of NMO-IgG to AQP4 in **(B)** and **(C)**, no pathology was observed after 3 days (n = 5 eyes).

Motivated by the success in creating NMO pathology in mouse brain by passive transfer of NMO-IgG and human complement by intracerebral injection [23], several approaches were tried to create ON or retinal cytotoxicity in mice. We first delivered NMO-IgG and complement to the anterior optic nerve by injection approximately 2 mm lateral to the anterior optic nerve following its exposure by lateral canthotomy. Figure 1A shows strong AQP4 expression in the anterior optic nerve, but only extradural deposition of NMO-IgG in surrounding skeletal muscle and soft tissue, as revealed using a fluorescently labeled anti-human secondary antibody (NMO-IgG is a human antibody). Attempts to increase contact time by using viscous gel vehicles were also unsuccessful in allowing access of NMO-IgG to optic nerve tissue, probably because of its dural ensheathment anterior to the optic canal.

We next tested intravitreal injection of NMO-IgG and complement to evaluate the potential for direct retinal injury, as AQP4 is strongly expressed on retinal Müller cells [31], and NMO-associated retinal abnormalities have been described [32-34]. As shown in Figure 1B, NMO-IgG binding to the AQP4-expressing retinal Müller cells was detected with a secondary anti-human antibody. No binding of a control (non-NMO) IgG was seen. However no pathology was observed in the retina as judged by preserved AQP4 immunofluorescence (Müller cell marker), absence of CD45-positive inflammatory cells, and deposition of activated complement (C9neo immunostaining) (not shown). Also, NMO-IgG staining was absent in the optic nerve. We speculate that the absence of retinal pathology may be due to limited access of intravitreally injected complement proteins beyond the inner blood-retina barrier to Müller cells and/or their inactivation by soluble inhibitors of complement in vitreous fluid or retinal tissue.

Reasoning that the posterior optic nerve and optic chiasm lack the dura that encases the anterior optic nerve, we tested the delivery of NMO-IgG and complement by a single injection near the optic chiasm by an intracranial route, using stereotaxic methodology and a head-bobbing sign to indicate needle contact with the cranial floor underlying the optic chiasm/posterior nerves (see Methods). The accuracy of needle placement was confirmed in preliminary studies in which dye-containing gels were injected and the optic nerves visualized following craniectomy. Figure 1C shows staining of the optic nerve periphery with NMO-IgG, with no staining by a control (non-NMO) IgG. While brain parenchyma near the optic nerve showed marked NMO pathology, little or no optic nerve pathology was seen, as evidenced by preservation of AQP4, GFAP and MBP (myelin) immunofluorescence, and absence of CD45-positive infiltrating leukocytes (not shown).

### Continuous perichiasmal infusion of neuromyelitis optica immunoglobulin G antibody and complement produces neuromyelitis optica optic neuritis

**Figure 2:**
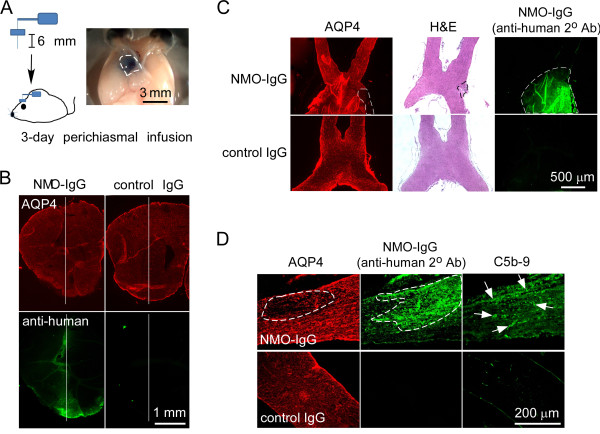
**Continuous perichiasmal infusion of neuromyelitis optica immunoglobulin G antibody and complement leads to aquaporin-4 loss and complement activation in optic nerve. (A)** (Left) Three-day continuous intracranial perichiasmal infusion of neuromyelitis optica immunoglobulin G antibody (NMO-IgG) and human complement was done by implantation of an osmostic pump. (Right) Area of diffusion in optic chiasm and brain of an infused blue dye (dashed line). **(B)** Immunofluorescence showing binding of NMO-IgG to aquaporin-4 (AQP4) in brain around the needle tract (white line) at 3-days after infusion of NMO-IgG or control IgG and complement. **(C)** AQP4 immunofluorescence, hematoxylin and eosin (H&E) staining and NMO-IgG immunofluorescence of the optic nerve. Dashed lines show lesion or area of NMO-IgG deposition. **(D)** Immunofluorescence showing AQP4 loss, NMO-IgG deposition and complement activation (C5b-9, arrows) in the optic nerve after infusion of NMO-IgG as in **(A)**. White dashed lines demarcate the lesion.

We speculated that the absence of NMO pathology following a single perichiasmal injection of NMO-IgG and complement may be related to minimal contact time of the injected macromolecules with the optic nerve, as their diffusion in brain extracellular space is likely much faster than along the parallel white matter tracts of the optic nerve [35,36]. In order to promote sustained optic nerve exposure, NMO-IgG and complement were delivered continuously over 3 days using an implanted mini-pump with needle tip placement near the optic chiasm. Figure 2A shows Evan’s blue dye distribution following 3-days mini-pump infusion, which includes areas of posterior prechiasmal optic nerves, optic chiasm, optic tract and surrounding brain parenchyma. Figure 2B shows NMO-IgG binding to AQP4 in the brain parenchyma surrounding the needle tract. Figure 2C,D shows substantial NMO-IgG deposition in optic nerves, with focal loss of AQP4 immunoreactivity, cell infiltration, and deposition of activated complement, indicating access of NMO-IgG and complement to the optic nerve, with development of NMO-like pathology. No lesions were seen following infusion of a control IgG and human complement.

**Figure 3:**
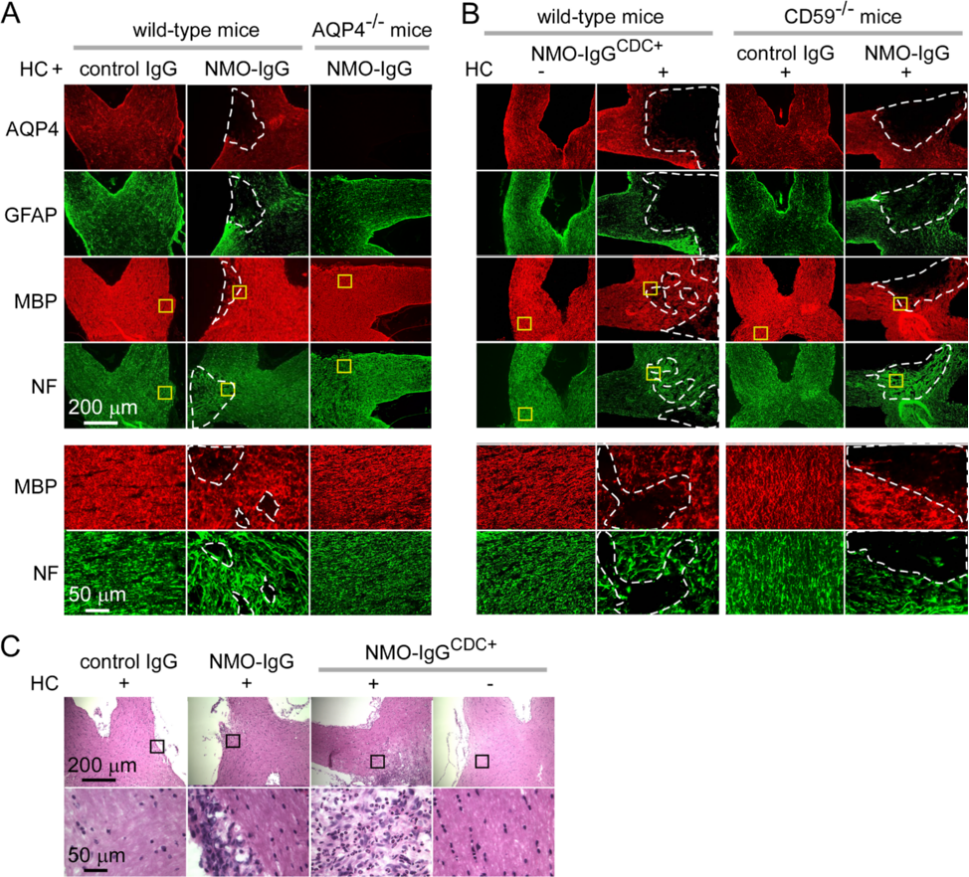
**Continuous perichiasmal infusion of neuromyelitis optica immunoglobulin G antibody and complement produces complement-dependent, neuromyelitis optica-like optic neuritis. (A)** (Top) Micrographs showing aquaporin-4 (AQP4), glial fibrillary acidic protein (GFAP), myelin basic protein (MBP) and neurofilament (NF) immunoreactivity in the optic nerve after 3-days continuous perichiasmal infusion of complement with neuromyelitis optica immunoglobulin G antibody (NMO-IgG) or control IgG in wild-type mice and NMO-IgG in AQP4^-/-^ mice. White dashed line demarcates area with loss of immunofluorescence. (Bottom) Higher magnification of yellow boxes. **(B)** (Top) AQP4, GFAP, MBP and NF immunofluorescence in the optic nerve after 3-days continuous perichiasmal infusion of (left) NMO-IgG^CDC+^ with or without complement in wild-type mice or (right) complement with control IgG or NMO-IgG in CD59^-/-^ mice. White dashed line demarcates region with loss of immunofluorescence. (Bottom) Higher magnification of yellow boxes. **(C)** Hematoxylin and eosin staining of the optic nerve after 3-days continuous perichiasmal infusion in wild-type mice of control IgG or NMO-IgG or NMO-IgG^CDC+^, with or without complement. HC, human complement.

Optic nerves were analyzed in 12 mice receiving a 3-day infusion of NMO-IgG and complement and 10 mice receiving the same quantity of control (non-NMO) IgG and complement. Eight out of 12 mice receiving NMO-IgG and complement showed characteristic NMO pathology in the optic nerve, with focal reductions in AQP4, GFAP, MBP and neurofilament immunofluorescence (Figure 3A). The precise site of the pathology was variable, likely reflecting slight variation in the location of the needle tip. None of the 10 mice receiving control IgG and complement developed pathology, nor did any of five mice receiving NMO-IgG alone (not shown), or of five AQP4 knockout mice receiving NMO-IgG and complement (Figure 3A). Development of optic nerve pathology therefore requires NMO-IgG and complement, as well as AQP4 expression.

Based on the central role of complement in NMO pathogenesis, we reasoned that increased complement activity would result in more profound optic nerve pathology. Two approaches were tested: (a) passive transfer of a mutated recombinant NMO-IgG (NMO-IgG^CDC+^) having ~10-fold increased complement effector function [26]; and (b) infusion in mice lacking CD59, the major membrane-associated complement inhibitor protein on mouse astrocytes [37,38]. Robust and more widespread NMO pathology was seen following 3-days infusion of NMO-IgG^CDC+^ and complement in wild-type mice, and of (non-mutated) NMO-IgG and complement in CD59-null mice (Figure 3B).

One of the characteristics of NMO pathology is inflammatory cell infiltration. After 3-days infusion with NMO-IgG or NMO-IgG^CDC+^ and complement, optic nerves showed inflammatory cell infiltration on hematoxylin and eosin staining (Figure 3C), mainly mononuclear inflammatory cells. Inflammatory cell infiltration was not seen with control IgG with complement, NMO-IgG^CDC+^ alone, or NMO-IgG with complement in AQP4^-/-^ mice (data not shown).

### Characterization of optic neuritis pathology produced by infusion of neuromyelitis optica immunoglobulin G antibody

**Figure 4:**
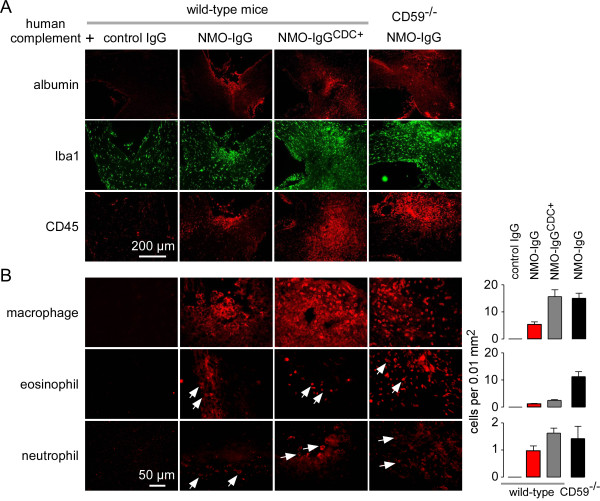
**Inflammation in optic nerve lesions. (A)** Immunostaining for albumin, ionized calcium-binding adaptor molecule-1 (Iba1); microglia and CD45 (leukocytes) in the optic nerve after 3-days continuous perichiasmal infusion of neuromyelitis optica immunoglobulin G antibody (NMO-IgG) and complement. **(B)** (Left) Immunostaining with markers for macrophages, neutrophils and eosinophils. (Right) Number of infiltrating cells of each per 0.01 mm^2^ (mean ± SEM, n = 3).

The characteristic features of NMO pathology include AQP4, GFAP, myelin loss and inflammatory cell infiltration, as shown above, as well as BBB disruption, and inflammation with microglial activation and macrophage and granulocyte infiltration [6-8]. Figure 4A shows focal albumin extravasation in areas of NMO pathology, demonstrating BBB leakage. Localized inflammation was also evident with increased Iba1 (microglial marker) and CD45 (leukocyte) immunofluorescence (Figure 4A). Albumin extravasation and inflammation were absent in mice receiving a 3-day infusion of control IgG and complement, and increased in wild-type mice receiving NMO-IgG^CDC+^ and complement, and in CD59-null mice receiving NMO-IgG and complement.

The composition of the CD45-positive cell infiltrate was determined using antibodies against cell-specific markers. Figure 4B shows positive immunofluorescence for macrophages (F4/80), eosinophils (Siglec-F) and neutrophils (Ly-6G), with quantification showing a greater number of macrophages than granulocytes in this model. Cell-specific immunofluorescence was absent in mice receiving a 3-day infusion of control IgG and complement.

### Retinal ganglion cell loss

**Figure 5:**
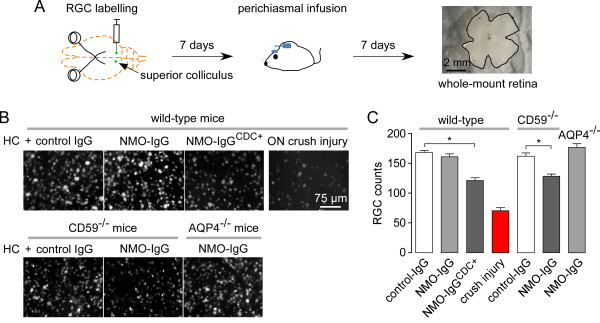
**Continuous perichiasmal infusion of neuromyelitis optica immunoglobulin G antibody and complement produces retinal ganglion cells loss. (A)** Retrograde labeling of retinal ganglion cells (RGCs) by injection of FluoroGold in the superior colliculus. After 7 days, neuromyelitis optica immunoglobulin G antibody (NMO-IgG) and complement were infused near the optic chiasm by minipump. Retinas were collected 7 days later. **(B)** Micrographs showing RGC loss. **(C)** Quantification of RGC number per 0.06 mm^2^ microscope field (mean ± SEM, n = 4, *P < 0.001). AQP4, aquaporin-4; HC, human complement; (ON, optic nerve).

A consequence of NMO ON is retrograde RGC loss leading to visual deficit. We quantified RGC number in whole-retina mounts at 7 days after delivery of NMO-IgG and human complement to posterior optic nerve (Figure 5A). Figure 5B,C shows no clear reduction in the number of RGCs in mice receiving a 3-day infusion of NMO-IgG and complement compared to control IgG. RGC number was reduced by 28% in wild-type mice administered NMO-IgG^CDC+^ and complement and by 21% in CD59^-/-^ mice injected with NMO-IgG. No RGC loss was seen in AQP4^-/-^ mice injected with NMO-IgG. Optic nerve crush injury was used as a positive control of RGC damage.

## Discussion

Current understanding of NMO pathogenesis comes largely from data on brain and spinal cord, as there is little descriptive pathology of optic nerves in human NMO and adequate animal models of NMO ON have not been developed. The bulk of evidence supports a pathogenesis mechanism that involves NMO-IgG access to the central nervous system and binding to AQP4 on astrocytes, which causes complement- and cell-mediated cytotoxicity [22,26,39-42]. The primary astrocyte damage initiates an inflammatory reaction with cytokine release, granulocyte and macrophage infiltration, and further BBB disruption, which produces secondary oligodendrocyte injury, demyelination and neuron loss. This mechanism is supported by pathology in human spinal cord and brain [43,44], by brain pathology in mice following passive transfer of NMO-IgG by intracerebral injection [23], and by *ex vivo* studies in spinal cord slice cultures exposed to NMO-IgG and various effector molecules and cells [45]. It has been assumed without direct evidence that a similar pathogenesis mechanism applies to NMO ON. Studies done in *ex vivo* optic nerve cultures exposed to NMO-IgG and complement showed an astrocytopathy with demyelination, but this model is limited by the short-term (~1 day) viability of optic nerve cultures [45].

The main finding of the study here is that passive transfer of NMO-IgG and human complement to mice by continuous intracranial infusion near the optic chiasm produces optic nerve lesions with loss of AQP4 and GFAP immunoreactivity, demyelination, and inflammation with prominent macrophage infiltration. Optic nerve pathology was exacerbated when NMO-IgG was administered to CD59 knockout mice or when a mutated NMO antibody with enhanced complement effector function was administered to wild-type mice, supporting a central role of complement in NMO ON in our model. Control studies, including infusions in AQP4 knockout mice, indicated that the pathological changes require NMO-IgG, complement and AQP4. The passive transfer model of NMO ON established here should be useful in studying mechanisms of NMO pathogenesis specific to the anterior visual pathway, as well as in evaluating potential vision-preserving therapies using clinically relevant *in vivo* anatomic and functional outcome measures.

Prior models of general neuroinflammation include optic nerve pathology, though the pathogenesis mechanisms are very different from that of NMO, which involves a humorally mediated astrocytopathy. As mentioned in the Introduction, ON is a well-described feature of experimental autoimmune encephalomyelitis. ON has also been seen in a subpopulation of transgenic mice expressing myelin oligodendrocyte glycoprotein (MOG)-specific T cell receptors [46]. Crossing this mouse with a MOG-specific Ig heavy-chain knock-in mouse produced mice with both T and B cell MOG reactivity [29]. This double transgenic mouse manifests selective optic nerve and spinal cord pathology (with 60% penetrance) and a Th17 differentiation bias, reminiscent of NMO [47]. While ON is seen in these various models, they are probably not useful to study NMO pathogenesis mechanisms or test NMO therapeutics.

Several approaches to deliver NMO-IgG and complement were tested to produce robust ON in mice. Delivery of NMO-IgG and complement to the anterior optic nerve following lateral canthotomy did not produce optic nerve pathology, probably because of limited access of infused macromolecules to the ensheathed anterior optic nerve. Though intravitreal injection of NMO-IgG and complement resulted in NMO-IgG binding to AQP4 in retinal Müller cells, no retinal pathology was seen, perhaps because of limited access of some complement components to inner retinal layers. Also, intravitreally delivered solutes and macromolecules are unable to diffuse into the optic nerve. From these initial studies, we postulated that delivery of NMO-IgG and complement near the unsheathed perichiasmal posterior optic nerve might produce NMO ON. Though a single intracerebral injection of NMO-IgG and complement with the needle tip near the optic chiasm resulted in some NMO-IgG binding to optic nerve, as well as NMO pathology in brain near the optic nerve, little NMO pathology was found. Robust NMO pathology required continuous intracerebral injection of NMO-IgG and complement near the optic nerve. We conclude that the sustained exposure of target tissues to pathogenic macromolecules afforded by continuous infusion better recapitulates human NMO in which progressive pathology involves an amplifying cycle of astrocyte cytotoxicity, inflammation and BBB disruption. We cannot exclude, however, the possibility that the prechiasmal optic nerve and optic chiasm may be more susceptible to NMO-IgG-mediated pathology than the anterior optic nerve.

Though the NMO ON model developed here produced robust lesions with characteristic NMO pathology, there are a number of limitations of the model and potential directions for future advances. Continuous intracerebral infusion with precise needle placement is invasive and technically challenging. The direct administration of human complement, which was necessary because of the weak activity of mouse complement and the presence of complement inhibitory factor(s) in mouse serum [48], does not accurately recapitulate the human disease in which endogenous complement proteins derive primarily from the serum. We recently established a rat model of NMO involving intracerebral administration of NMO-IgG without added complement, which produced robust NMO pathology in brain around the needle track [16]. Pathology required active rat complement, as complement inactivation by cobra venom factor prevented the astrocyte cytotoxicity and demyelination. In a recent variation of the rat model, NMO pathology was produced in brain by peripheral NMO-IgG administration and focal mechanical BBB disruption (Asavapanumas and colleagues, unpublished results). Models of NMO ON in rats involving peripheral or optic nerve-targeted NMO-IgG delivery, perhaps in combination with maneuvers to disrupt the blood-optic nerve barrier, may produce ON without the need to administer complement.

It remains unclear why NMO pathology is primarily restricted to optic nerve and spinal cord, and to a lesser extent in brain, with little or no pathology in peripheral AQP4-expressing tissues. Optic nerve susceptibility in NMO is unlikely due solely to NMO-IgG access, and may involve impaired diffusion of NMO-IgG, soluble pro-inflammatory factors and complement proteins from focal areas of central nervous system entry. Optic nerve susceptibility in NMO might also arise from the high AQP4 expression in the optic nerve compared to brain [47] and the abundance of large orthogonal arrays of particles in perivascular astrocytic end-feet of the optic nerve [49-51] that promote tight binding of AQP4-IgG and efficient CDC [52]. Plasmablasts in the cerebrospinal fluid secreting NMO-IgG locally [22] and/or regional variations in the expression of complement regulator or key BBB proteins may also play a role.

## Conclusions

Passive transfer of NMO-IgG, when supplemented with human complement, produced ON in mice with characteristic NMO pathology. Development of robust pathology required continuous exposure of the posterior optic nerve to NMO-IgG and complement, which was accomplished by 3-days intercerebral infusion using an implanted mini-pump with needle positioned near the optic chiasm. Our data show that, as found in brain, passive transfer of NMO-IgG and complement is sufficient to produce ON in mice. NMO ON models should be useful in testing novel disease-modifying therapeutics aimed at preserving vision.

## Abbreviations

AQP4: aquaporin-4; BBB: blood–brain barrier; GFAP: glial fibrillary acidic protein; Iba1: ionized calcium-binding adaptor molecule-1; IgG: immunoglobulin G; MBP: myelin basic protein; MOG: myelin oligodendrocyte glycoprotein; NMO: neuromyelitis optica; NMO-IgG: neuromyelitis optica immunoglobulin G antibody; ON: optic neuritis; RGC: retinal ganglion cell; UCSF: University of California, San Francisco.

## Competing interests

The authors declare that they have no competing interests.

## Authors’ contributions

NA and JR carried out experimental work and wrote the manuscript draft. MCP, JLB, MHL and ASV designed experiments and edited the manuscript. All authors read and approved the final manuscript.
